# Hashimoto’s encephalopathy cases: Chinese experience

**DOI:** 10.1186/1471-2377-12-60

**Published:** 2012-07-24

**Authors:** Yi Tang, Yi Xing, Michael T Lin, Jin Zhang, Jianping Jia

**Affiliations:** 1 Department of Neurology, Xuan Wu Hospital, Capital Medical University, 45 Changchun Street, Beijing, 100053, People’s Republic of China; 2 Department of Neurology and Neuroscience, Weill Medical College of Cornell University/New York-Presbyterian Hospital, New York, USA

## Abstract

**Background:**

Hashimoto’s encephalopathy is a poorly understood syndrome consisting of heterogeneous neurological symptoms and high serum antithyroid antibody titers, typically responding to steroids. More clinical series studies are required to characterize the clinical, laboratory and imaging features, and outcomes, especially in the Chinese population.

**Methods:**

We analyzed the clinical, laboratory, and imaging features and outcomes of thirteen consecutive patients with Hashimoto’s encephalopathy diagnosed in Xuan Wu Hospital, Beijing from 2005 to 2010 retrospectively.

**Results:**

Cognitive impairment (84.6%) and psychiatric symptoms (38.5%) were the most frequent symptoms. Seizures (30.8%) and myoclonus (7.7%) were less common than previously described. Three (23.1%) patients showed abnormal signals in hippocampus or temporal lobe, which were believed related to their memory disorders or seizures. MRI changes showed resolution paralleling clinical improvement in one patient. Among eight patients who received steroid therapy, five patients recovered, one patient improved with residual deficits, and two patients relapsed or had no effect. Among five non-steroid treated patients, three patients experienced stable remission with antiepileptic drugs or general neurotrophic therapy, and two patients experienced continuous deterioration.

**Conclusions:**

Most patients with Hashimoto’s encephalopathy showed good response to steroids. Some patients improved without steroid therapy. Considering its reversible course, we recommend that Hashimoto’s encephalopathy should always be in the differential diagnosis while evaluating disorders of the central nervous system.

## Background

Hashimoto’s encephalopathy (HE), also known as steroid-responsive encephalopathy associated with autoimmune thyroiditis (SREAT) [1], is a rare neurological syndrome that is poorly understood and often misdiagnosed. The first case was described by Lord Brain in 1966, in which a patient with Hashimoto thyroiditis (HT) showed impairment of consciousness, tremor, cognitive loss and stroke-like episodes [2]. The clinical manifestations of HE include cognitive impairment, transient aphasia, tremor, myoclonus, ataxia, seizure, sleep disturbance and headache, with fluctuating symptoms in 95% of patients [1]. Two subtypes were proposed: a vasculitic type with stroke-like episodes and a diffuse, progressive type with insidious onset but progressive deterioration of mental functions [3]. HE is generally considered to be an autoimmune encephalopathy. However, the pathogenesis is still not clear. Anti-thyroid peroxidase antibody (TPO-Ab), present in almost all HE cases [4], can also be found in general population with euthyroid status [5]. A direct causal relationship between thyroid antibodies and HE seems unlikely [3].

The first set of diagnostic criteria for HE was put forward by Peschen-Rosin and co-workers in 1999, which encompassed unexplained episodes of relapsing myocloni, generalized seizures, focal neurological deficits or psychiatric disorders, and at least 3 of the following: abnormal EEG, elevated thyroid antibodies, elevated CSF protein and/or oligoclonal bands, excellent response to steroids, unrevealing cerebral MRI [6]. After that, the knowledge of symptoms of HE was much more widened. Good steroid response was not observed in all HE patients [7-10]. Although the role in the pathogenesis of HE is still debated, it can’t be ignored that the elevation of anti-thyroid antibodies was observed in all HE patients. Therefore, the following principles were included in the diagnostic criteria by majority of researchers [1,7,8]: the elevated serum antithyroid antibodies; neurological illness mostly presented as clouding of consciousness, cognitive impairment, seizures, myoclonus, ataxia, psychiatric symptoms and focal neurological deficits; exclusion of infectious, toxic, metabolic, vascular or neoplastic etiologies.

Because of the low prevalence (about 2.1/100 000) [11], varied clinical features, unclear pathogenesis and histopathologic characteristics [1,8,12], currently no recognized diagnostic criteria for HE have been established. Therefore, more clinical series studies are required to characterize the clinical, laboratory and imaging features, and outcomes of HE patients. To our knowledge, there has been no published series report of HE from Asia yet. In this study, we try to characterize the clinical features and outcomes of a series of Chinese HE cases in whom the diagnosis of HE was made at Xuan Wu Hospital (in Beijing) between 2005 and 2010. The inclusion criteria for this study are: (1) encephalopathy manifested by clouding of consciousness, cognitive impairment, seizures or neuropsychiatric features; (2) elevated anti-TPO antibody with euthyroid status (serum sensitive thyroid-stimulating hormone [TSH], 0.35–5.5 uIU/ml); (3) no alternative infectious, toxic, metabolic, vascular or neoplastic etiology related to the neurological symptoms in blood, urine, cerebrospinal fluid (CSF) or neuroimaging examinations.

## Methods

This retrospective study received approval from the Xuan Wu Hospital institutional review board. We searched the electronic medical record system to identify and review patients diagnosed with HE at Xuan Wu Hospital (in Beijing) between 2005 and 2010, using the following criteria: (1) encephalopathy manifested by clouding of consciousness, cognitive impairment, seizures or neuropsychiatric features; (2) elevated anti-TPO antibody with euthyroid status (serum sensitive thyroid-stimulating hormone [TSH], 0.35–5.5 uIU/ml); (3) no alternative infectious, toxic, metabolic, vascular or neoplastic etiology related to the neurological symptoms in blood, urine, cerebrospinal fluid (CSF) or neuroimaging examinations. We did not include steroid response as a criterion because not all HE patients respond to corticosteroid therapy [7,8,10]. Second, corticosteroids were not the standard therapy for HE in our institution before 2008, and some patients were unwilling to take the risks of steroid therapy. These patients provided the opportunity to observe the clinical course of HE without steroid therapy. Furthermore, elevated serum anti-TPO antibody was included as a diagnostic criterion for its much higher prevalence in HE patients than anti-thyroglobulin (anti-TG) antibody [1,4,8].

After identification of patients, demographic, clinical, laboratory, imaging data and treatment responses were collected. Furthermore, we followed up all patients and present the long-term clinical course of HE.

## Results

### Demographic and clinical data

Thirteen patients were identified. The mean age at onset was 42 years (range, 21 to 63 years), and 10 were female. Three patients had a history of thyroid disorders. One patient had systemic lupus erythematosus, and another had Raynaud’s syndrome. Eleven patients (84.6%) had been misdiagnosed as other disorders, including stroke or transient ischemic attack (23.1%), demyelinating disease (15.4%), viral encephalitis (15.4%), mitochondrial encephalopathy (7.7%), essential tremor (7.7%), Alzheimer’s disease (7.7%), and paraneoplastic neurological syndrome (7.7%). Clinical presentations are summarized in Table 1. The most common symptom was cognitive impairment (11 patients, 84.6%). Among these patients, 9 had complaints of cognitive deficits (Patients No. 1, 4, 5, 6, 8, 10, 11, 12, and 13), which had been confirmed by objective examination, and 2 were found to have cognitive impairments through neurological examination (Patients No. 3 and 7). The memory impairment was the predominant symptom in majority of these patients (Patients No. 1, 4, 5, 8, 10, 11 and 13), and others had multiple cognitive domains impairment (Patients No. 3, 6, 7 and 12), including memory, calculation, orientation and language. The cognitive abilities of these patients were evaluated by at least one cognitive scale (Table 1), including the Mini-Mental State Examination (MMSE) [13], the Clinical Memory Scale (CMS) [14] and the Wechsler Adult Intelligence Scale-Revise Chinese (WAIS-RC) [14], except for patient No. 6, who could not cooperate with the detailed cognitive tests on admission. Other common symptoms included psychiatric symptoms (5 patients, 38.5%), seizures (4 patients, 30.8%) and sleep disturbance (4 patients, 30.8%). Three patients (23.1%) manifested with stroke-like episodes. Dizziness and headache were each present in 2 (15.4%) patients. Myoclonus, ataxia or gait disorder, tremor, aphasia and lateralized sensory deficit, were all less common (1 patient each, 7.7%).

**Table 1:** Clinical findings and treatment outcomes of 13 patients with Hashimoto’s Encephalopathy

**NO.**	**Sex**	**Age (year)**	**Duration**	**Clinical manifestations**	**Cognitive tests**	**Antibodies, U/ml (before treatment)**	**CSF protein**	**MRI**	**EEG**	**Treatment**	**Antibodies, U/ml (after treatment)**	**Follow-up period (month)**	**Clinical course**
1	F	52	19 months	Memory impairment, psychiatric symptoms, insomnia	MMSE, 21	TPO>1300	+	+		Levothyroxine		22	No improvement
						TG>500							
2	F	32	6 months	Generalized seizures		TPO>1300	+	−	++	Antiepileptic treatment		12	No further seizures
						TG>500							
3	M	58	1 year	Recurrent stroke-like episodes, multiple cognitive domains impairment, psychiatric symptoms	MMSE, 20	TPO 831.9	+	−	++	Intravenous methylprednisolone 500 mg for 3 d followed by oral prednisone taper	TPO 492.0	13	Two stroke-like episodes
						TG 41.9					TG 40.7		
4	M	27	3 weeks	Memory impairment	MMSE, 25	TPO>1300	+	+	−	Nerve growth factor, vitamin B12		15	Return to normal cognitive function, MMSE, 30
						TG 38.8							
5	F	29	6 years	Memory impairment, psychiatric symptoms, aphasia, seizures, myoclonus	CMS, 47	TPO 519.1	−	+		Intravenous methylprednisolone 1 g for 5 d		20	No improvement
						TG 114.7							
6	F	46	3 days	Stroke-like episode, multiple cognitive domains impairment, hypersomnolence, headache		TPO 557.8	−	+	−	Intravenous methylprednisolone 1 g for 5 d followed by oral prednisone taper	TPO 273.8	22	Complete remission, no further stroke-like episode
						TG 44.3					TG 40.2		
7	M	63	7 days	Acute headache, multiple cognitive domains impairment	MMSE, 17	TPO>1300	−	+	+	Intravenous methylprednisolone 1 g for 5 d		17	Complete remission, no headache, MMSE, 24
						TG 122.6							
8	F	43	2 months	Ataxia, memory impairment, seizures	MMSE, 19	TPO >1300	+	−	++	Intravenous methylprednisolone 1 g for 5 d		13	Complete remission, no further seizures, MMSE, 26
						TG 115.9							
9	F	21	2 months	Generalized seizures		TPO >1300	+	+	++	Antiepileptic drugs		9	No further seizures
						TG 37.9							
10	F	40	1 month	Dizziness, memory impairment, psychiatric symptoms, hypersomnolence	CMS, 72	TPO 139.6	−	+		Intravenous methylprednisolone 500 mg for 5 d followed by oral prednisone tapering		25	Complete remission, no dizziness and hypersomnolence, CMS, 91
						TG 24.2							
11	F	37	2 years	Memory impairment, tremor	CMS, 68	TPO>1300	−	−	−	Nerve growth factor, vitamin B12		20	Continue progression
						TG 104.9							
12	F	50	4 years	Multiple cognitive domains impairment, insomnia, psychiatric symptoms	MMSE, 9	TPO >1300	+	+	+	Intravenous dexamethasone, 10 mg for 2 weeks, followed by oral methylprednisolone taper		19	Partial improvement, MMSE, 16
						TG 46.9							
13	F	43	3 months	Stroke-like episode, lateralized sensory deficits, dizziness, memory impairment	WAIS-RC, 84	TPO >1300	+	+	+	Intravenous methylprednisolone 500 mg for 5 d followed by oral prednisone taper	TPO 1144.8	18	Complete remission, no further stroke-like episode
						TG 45.6					TG 48.4		

Abbreviations: TPO, thyroid peroxidase; TG, thyroglobulin; MMSE, Mini-Mental State Examination; CMS, Clinical Memory Scale; WAIS-RC, Wechsler Adult Intelligence Scale-Revise Chinese; MRC, Medical Research Council.

CSF protein: −, normal; +, elevated protein levels; MRI: −, normal; +, abnormal signals or cerebral atrophy; EEG: −, normal; +, generalized slowing; ++, epileptiform abnormalities.

### Serologic and CSF studies

The upper detectable limits of anti-TPO antibody and anti-TG antibody tests in our institution were 1300 U/ml and 500 U/ml respectively. Test values higher than the upper limits were regarded as 1300 U/ml and 500 U/ml in the present study. By definition, elevated TPO-Ab (average, 1057.6 U/ml; range, 139.6–1300 U/ml; normal reference range, 0–60 U/ml) and euthyroid status were observed in all patients. The serum level of anti-TG antibody was elevated in 6 (46.2%) patients (average, 133.7 U/ml; range, 24.2–500 U/ml; normal reference range, 0–60 U/ml). No CSF pleocytosis was observed. The CSF protein level was elevated in 8 (61.5%) patients (average, 50.4 mg/dl; range, 22–146 mg/dl; reference range, 15–45 mg/dl). Other serologic and CSF findings were given in Table 2.

**Table 2:** Laboratory findings of 13 patients with Hashimoto’s Encephalopathy

**Test**	**Number (%) of patients/total number of patients tested**
Serologic findings	
Anti-TPO antibody elevated	13 (100)/13
Anti-TG antibody elevated	6 (46.2)/13
Aminotransferase level elevated	5 (38.5)/13
gG level reduced	3 (23.1)/13
Complement C3 level reduced	3 (23.1)/13
ANA positive	2 (28.6)/7
ENA antibody positive	2 (18.2)/11
Anti-SSA antibody	1 (9.1)/11
Anti-Jo-1 antibody	1 (9.1)/11
C reactive protein level elevated	1 (7.7)/13
Rheumatoid factor positive	1 (7.7)/13
CSF	
Protein levels elevated	8 (61.5)/13
Myelin basic protein levels elevated	3 (33.3)/9
gG synthesis rate elevated	3 (33.3)/9
Oligoclonal bands positive	1 (11.1)/9

Abbreviations: TPO, thyroid peroxidase; TG, thyroglobulin; ESR, erythrocyte sedimentation rate; ANA, antinuclear antibody; ENA, extractable nuclear antigen; ANCA, anti-neutrophil cytoplasmic antibody; ACL, anticardiolipin.

## Electroencephalogram (EEG)

EEG was tested in 10 patients. Three (30%) were normal; 4 (40%) had epileptiform abnormalities (Patients No. 2, 3, 8 and 9) and 3 (30%) showed generalized slowing (Patients No. 7, 12 and 13).

## Magnetic resonance imaging (MRI)

**Figure 1:**
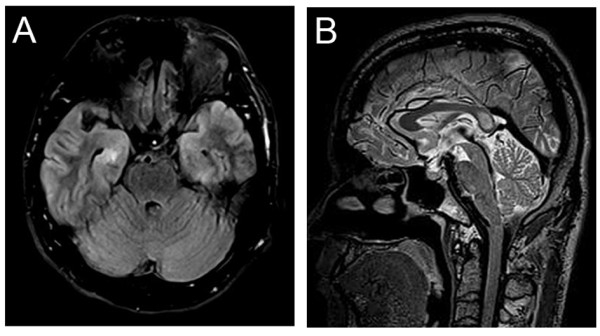
**MRI scans of HE patients.** (**A** and **B**) Patient No.4 with memory impairment. The splenium corporis callosi and bilaterial hippocampal abnormalities are believed to be responsible for the memory impairment.

All 13 patients underwent brain MRI examination. Four (30.8%) patients (Patients No. 2, 3, 8 and 11) had normal results. Nonspecific abnormal signals in white matter were noted in 6 (46.2%) patients (Patients No. 1, 5, 6, 7, 12 and 13). One (7.7%) patient (Patient No. 5) had cerebral atrophy. Three (23.1%) patients (Patients No. 4, 9 and 10) had hippocampus, temporal lobe or splenium corporis callosi abnormal signals (Figure 1), which were believed related to their memory disorders or seizures. One patient (Patient No. 6) showed complete resolution in MRI follow-up.

## Treatment and follow-up

We followed up all 13 patients (average, 14.5 months; range, 9–25 months). The detailed treatments, responses and related data were given in Table 1.

Among the 8 patients (61.5%) who received corticosteroid therapy, 5 (62.5%) had complete remission; 1 (12.5%) had partial improvement; 1 (12.5%) had relapses, and 1 (12.5%) had no response in our follow-up. Serum levels of antithyroid antibodies were reduced in all the 3 patients retested after corticosteroid treatment. The outcomes of steroid treatment were not associated with the serum levels of antithyroid antibodies, while patients who received early treatment were more likely to have complete remission, especially for patients with stroke-like episodes. For patients whose symptoms persisted for a long time before steroid therapy, it seemed difficult to get significant improvement.

Among 5 patients not treated with steroids, 2 patients with seizures were treated with antiepileptic drugs and had no further epileptic attacks over 12 and 9 months of follow up. No improvement was observed in 1 patient treated with levothyroxine. With general neurotrophic therapy, including nerve growth factor and vitamin B12, complete remission was observed in 1 patient and continued deterioration in the other patient.

## Discussion

HE has attracted growing attention in the last 10 years. This is partly due to its treatability and unclear pathogenesis. In China, HE is still an under recognized entity because of its various clinical manifestations and absence of specific biomarkers. In this retrospective study, by analyzing the clinical, laboratory, imaging features and outcomes of 13 Chinese HE patients, we try to provide more characterization of HE, especially in Chinese patients.

The clinical symptoms of our patients are quite varied. Cognitive impairment and psychiatric symptoms are among the most common symptoms. However, the frequency of seizures and myoclonus showed a big difference from previous reports. In the review by Ferracci et al., seizures and myoclonus are among the most frequent events in all 121 reported HE patients [7]. In other two studies, seizures and myoclonus are also ranked as common symptoms [1,8]. In our study, seizures were observed in 4 patients and myoclonus was observed in only 1 patient. Due to the various clinical manifestations, initial alternative diagnoses are not surprising. The most common misdiagnoses were ischemic cerebrovascular disease, demyelinating disease and viral encephalitis. Interestingly, unlike the study by Castillo et al. [1], neurodegenerative diseases, including Creutzfeldt-Jakob disease and Alzheimer’s disease, were not frequently considered.

Various neuroimaging features have been reported in HE. MRI may show normal, diffuse cortical atrophy or localized increased T2 signal [7,15]. In this study, most patients had normal MRI or nonspecific abnormalities. In one patient with MRI follow-up, MRI changes showed resolution paralleling clinical improvement. Interestingly, 3 (23.1%) patients with memory disorders or seizures showed abnormal signals in hippocampus or temporal lobe. Instead of providing direct evidence for diagnosis, neuroimaging studies in HE patients are more important for exclusion of other possible neurological disorders.

Currently, steroid therapy is considered the first choice for HE patients if there are no contraindications [7]. Some researchers include a beneficial response to glucocorticoid therapy as a criterion for the diagnosis of HE [1]. In this study, among 8 patients who received steroid therapy, 5 patients recovered, 1 patient improved with residual deficits, and 2 patients relapsed or had no effect. It is worth noting that among 5 non-steroid treated patients, 3 patients showed stable remission with antiepileptic drugs or general neurotrophic therapy. The true efficacy of steroids remains uncertain without a treatment trial, which is extremely difficult in rare diseases like HE.

The pathogenic mechanism of HE is unknown. The histologic finding of perivascular lymphocytic infiltration [16], and the response to steroid and other immunomodulatory therapies suggests an autoimmune disorder. Some evidences support the hypothesis of autoimmune vasculitis. The SPECT study showed focal or generalized hypoperfusion which might be produced by vasculitic disruption of cerebral microvasculature [17]. Pathological study demonstrated perivascular lymphocytic infiltration [16]. More important, an autoantigen, α-enolase, was identified in HE patients, but not in the HT and normal controls [18,19]. α-enolase is an antigen of the thyroid and brain. It is concentrated in endothelial cells. However, the vasculitic hypothesis met many challenges, especially negative pathological findings in some HE patients [20,21]. Elevated antithyroid antibodies are generally considered necessary for the diagnosis. However, different from other autoimmune neurological disorders such as myasthenia gravis in which autoantibodies have definite mechanism [22], the role of antithyroid antibodies in the pathogenesis of HE is still not clear. Recently, Blanchin et al. reported that anti-TPO antibody from HE patients were able to bind cerebellar astrocytes in HE patients but not in HT patients [23]. This may support the role of anti-TPO antibody in the pathogenesis of HE. However, considering its wide presence in population [24], more investigations are needed to prove the pathogenic role of anti-TPO antibody.

The term “Hashimoto’s encephalopathy” was first put forward by Lord Brian in 1966 [2]. Since there is no evidence that antithyroid antibodies have a role in pathogenesis, this term could be misleading. Some investigators proposed the term “steroid-responsive encephalopathy associated with autoimmune thyroiditis (SREAT)” [1] to replace “Hashimoto’s encephalopathy”. However, not all HE patients respond to corticosteroid therapy [7,8,10]. It also seems inappropriate to name a disorder with treatment response to a nonspecific therapy. Therefore, as Drs. Chong and Rowland noted: “Until the pathogenesis is understood, the eponym (Hashimoto’s Encephalopathy) seems to be the most appropriate name for the condition because it links the only known identifier, a high serum concentration of antithyroid antibodies, to the encephalopathy” [8].

Our study has several limitations. First, some HE patients, who showed good response to steroids, were not included due to lack of CSF studies. Second, not all patients underwent the same tests and therapy. However, these patients also provide us with an opportunity to observe the clinical course of HE without steroid therapy. Third, data presented here are mostly clinical observations. None of these 13 patients underwent brain biopsy.

## Conclusions

This study summarized the clinical, laboratory, imaging features and outcomes of 13 Chinese HE patients. Most patients showed good response to steroids. Some patients improved without steroid therapy. Considering its reversible course, we recommend that HE should always be in the differential diagnosis while evaluating disorders of the central nervous system.

## Competing interests

The authors declare that they have no competing interests.

## Authors’ contributions

Study concept and design: Drs Jia and Tang. Acquisition of data: Drs Tang, Xing and Zhang. Analysis and interpretation of data: Drs Tang, Lin, and Xing. Drafting of the manuscript: Drs Tang and Xing. Critical revision of the manuscript for important intellectual content: Drs Tang, Xing, Lin, Zhang and Jia. Administrative, technical, and material support: Dr Xing. Study supervision: Drs Tang and Jia. All authors read and approved the final manuscript.

## Pre-publication history

The pre-publication history for this paper can be accessed here:

http://www.biomedcentral.com/1471-2377/12/60/prepub
